# Possible mechanisms of action of clarithromycin and its clinical application as a repurposing drug for treating multiple myeloma

**DOI:** 10.3332/ecancer.2020.1088

**Published:** 2020-08-18

**Authors:** Nobuo Takemori, Hong-Kean Ooi, Goro Imai, Kazuo Hoshino, Masanao Saio

**Affiliations:** 1 Department of Internal Medicine, Division of Hematology, Imai Hospital, Tanaka-cho 100, Ashikaga, Tochigi 326-0822, Japan; 2 Department of Veterinary Medicine, Azabu University, Fuchinobe 1-17-71, Sagamihara, Kanagawa 252-5201, Japan; 3 Department of Internal Medicine, Imai Hospital, Tanaka-cho 100, Ashikaga, Tochigi 326-0822, Japan; 4 Department of Surgery, Imai Hospital, Tanaka-cho 100, Ashikaga, Tochigi 326-0822, Japan; 5 Laboratory of Histopathology & Cytopathology, Department of Laboratory Sciences, Gunma University, Graduate School of Health Sciences, 39-22, 3-chome, Showa-machi, Maebashi, Gunma 371-8514, Japan; a https://orcid.org/0000-0001-9742-8385

**Keywords:** clarithromycin, effect, myeloma growth factors, multiple myeloma treatments, mechanisms of action, COVID-19

## Abstract

Clarithromycin (CAM), a semisynthetic macrolide antibiotic, is a widely used antibacterial drug. Recently, the efficacy of CAM as an add-on drug for treating multiple myeloma (MM) has been noted. Its effect on treating MM has been confirmed in combination chemotherapies that include CAM. However, a single treatment of CAM has no efficacy for treating MM. Many myeloma growth factors (MGFs) including interleukin (IL)-6 are known to be closely involved in the development of MM. CAM has been shown to suppress many MGFs, particularly IL-6. The possible mechanisms of action of CAM in treating MM have been suggested to include its immunomodulatory effect, autophagy inhibition, reversibility of drug resistance, steroid-sparing/enhancing effect and suppression of MGFs. In addition, MM is characterised by uncontrolled cell growth of monoclonal immunoglobulin (Ig)-producing neoplastic plasma cells. Large quantities of unfolded or misfolded Ig production may trigger considerable endoplasmic reticulum stress. Thus, MM is originally a fragile neoplasm particularly susceptible to autophagy-, proteasome- and histone deacetylase 6-inhibitors. Taken together, CAM plays an important role in MM treatments through its synergistic mechanisms.

In addition, CAM with its pleiotropic effects on cytokines including IL-6 and indirect antiviral effects might be worth a try for treating COVID-19.

## Background information on Clarithromycin (CAM)

Clarithromycin (CAM: Biaxin^®^) belongs to the 14-membered macrolide antibiotic family together with erythromycin and roxithromycin. The antibacterial effect of CAM is related to its capacity to inhibit protein synthesis in bacteria by binding to subunit 50S of the bacterial ribosome [1]. Kanoh and Rubin [2] reported that the 14- and 15-membered macrolides showed immunomodulatory properties but not the 16-membered ones. Following intestinal absorption, it has a fairly rapid first-pass metabolism in the liver. Its main metabolites are 14-(R)-hydroxy CAM, 14-(S)-hydroxy CAM and N-desmethyl CAM. 14-(R)-hydroxy CAM is an active metabolite that is also responsible for its anti-bacterial effect. CAM is acid stable and has a half-life of 5–7 hours with an oral dose of 500 mg administered every 12 hours. It is compatible with twice a day administration [3]. Ingestion of food increases CAM peak plasma concentration (C_max_) but does not affect the extent of CAM bioavailability. In non-fasting healthy human subjects, C_max_ is estimated to be 3–4 μg/mL with an oral dose of 500 mg administered every 8–12 hours according to the database of the Food and Drug Administration (FDA) (https://www.accessdata.fda.gov/drugsatfda_docs/label/2012/050662s044s050,50698s026s030,050775s015s019lbl.pdf). Recently, the efficacy of CAM as an add-on drug for treating multiple myeloma (MM) has been reported [3]. In this paper, the significance of CAM as an add-on drug in MM treatments, its effects on myeloma growth factors (MGFs) [4–7], and its mechanisms of action leading to the suppression of MM cell proliferation were discussed.

## Antineoplastic effects of 14-membered macrolides including CAM

The first experimental report suggesting the presence of the antineoplastic effect of macrolide (erythromycin) dates back to 1995. Hamada* et al* [8] experimentally demonstrated that the erythromycin induced cytotoxic macrophages in tumour-bearing mice and that these cytotoxic macrophages might work as one of the effector cells together with NK cells against the tumours transplanted in the mice. Clinically, Mikasa* et al* [9] first reported that long-term treatment with CAM significantly increased the median survival time of patients with advanced non-small cell lung cancer (NSCLC). Sakamoto *et al* [10] demonstrated that NK activity was significantly increased in patients with unresectable NSCLC after 1-month treatment with CAM. Furthermore, using Lewis lung carcinoma (LLC)-inoculated mice, they demonstrated that the administration of CAM after anticancer chemotherapy strongly inhibited tumour growth and significantly increased the NK activity. Recently, antineoplastic or immunomodulatory effects of CAM have again attracted attention in clinical fields. Several investigators reported that the CAM monotherapy was effective in treating hematologic malignancies including extranodal marginal zone B-cell lymphoma [11], mucosa-associated lymphoid tissue lymphoma [12, 13], follicular B-cell lymphoma [14, 15] and Hodgkin’s lymphoma [16].

****According to the reports by Zhang *et al* [17], Klein and Bataillie [4], Klein [5], Hallek *et al* [18], Mahtouk *et al* [6] and Musolino *et al* [7], the following factors and cytokines are listed as MGFs: insulin-like growth factor-1 (IGF-1), hepatocyte growth factor (HGF), macrophage inflammatory protein **α** (MIPα), growth/differentiation factor 15 (GDF15), pleiotrophin (PTN), brain-derived neurotrophic factor (BDNF), IL-6 family cytokines (IL-6, ciliary neurotrophic factor [CNTF], oncostatin M [OSM], leukaemia inhibitory factor [LIF], IL-11 and cardiotrophin-like cytokine factor 1 [CCLF1]), other cytokines (IL-1β, IL-10, IL-15, IL-16, IL-21, IL-22 and IL-23), TNF family members (B-cell activating factor [BAFF] and a proliferation-inducing ligand [APRIL]), transforming growth factor-β(TGF-β), granulocyte colony-stimulating factor (G-CSF), granulocyte macrophage colony-stimulating factor (GM-CSF), epidermal growth factor (EGF) family members (amphiregulin [AREG], heparin-binding EGF-like growth factor [HB-EGF] and neuregulin1~4 [NRG1~4]), basic fibroblast growth factor (bFGF), Wnt family members (Wnt5A/10B/16), Jagged family (Jag1/2) and VEGF/PDGF family members. The important MGFs and other factors which are positively or negatively influenced by CAM are shown in Table 1 and briefly described as follows.

### IL-1

IL-1 is a potent proinflammatory cytokine that functions as an endogenous pyrogen. IL-1 is mainly responsible for IL-6 production in the tumoural environment through prostaglandin E2. IL-1β gene and IL-1β protein, though less potent, are expressed in myeloma cells [19]. The bone marrow stromal cells (BMSCs) react with IL-1β to produce and secrete large quantities of IL-6, which in turn stimulate the survival and expansion of MM cells [7, 19, 20]. The suppression of IL-1β by CAM [21–30] (Table 1) might be related to MM cell reduction.

### IL-2

IL-2 plays an essential role in key functions of the immune system, which includes immuno-tolerance and immunity. It is mainly produced by activated CD4^+^ T-cells and activated CD8^+^ T-cells and to a lesser extent by activated dendritic cells, NK/NKT cells and macrophages. IL-2 mediates its effects by binding to IL-2-receptors, which are expressed by lymphocytes. IL-2 is known to strongly stimulate NK-cell and T-cell growth which consequently augments the cytolytic action, leading to enhanced cytotoxicity. Anti-tumour properties of IL-2 are mediated by the cellular immune system, but IL-2 itself has no direct effect on tumours [31]. CAM has been reported to suppress IL-2 production [30, 32–34] (Table 1). The suppression of IL-2 by CAM seems to be disadvantageous from the view point of cellular immunity. However, the effect of reduced IL-2 on MM cells has not yet been fully elucidated.

### IL-4

IL-4 plays an essential role in promoting Th2 cell differentiation. It is primarily produced by Th2 cells, NKT cells, mast cells and eosinophils. It is also known that IL-4 induces B-cell class switching to IgE. The relationship between IL-4 and MM cell growth was demonstrated by Herrmann *et al* [35]. They showed that recombinant human IL-4 blocked endogenous IL-6 synthesis in a dose-dependent fashion and caused a significant reduction of plasma cell growth that could be reversed by exogenous recombinant human IL-6. Hamada* et al* [36] reported that CAM induced IL-4-producing T-cells in the spleen of tumour-bearing mice. Considering the suppressive effect of IL-4 on MM cell proliferation [35], it is probable that the activation of IL-4 by CAM might lead to MM cell reduction.

### IL-5

IL-5 is produced by Th2 cells and mast cells and is known to stimulate B-cell growth and increase immunoglobulin secretion, primarily IgA. This cytokine is known to synergise with IL-6 to support myeloma cell proliferation [4, 37]. The suppression of IL-5 by CAM [29, 30, 33, 38] (Table 1) might result in the reduction of MM cell.

### IL-6

IL-6 is a pleiotropic proinflammatory cytokine involved in acute inflammatory responses, immune reactions, haematopoiesis and inflammation. IL-6 is generated by monocytes, endothelial cells, macrophages and fibroblasts in response to diverse stimuli (TNF-α, IL-1 and IL-17) during systemic inflammation [7]. Among the MGFs, IL-6 represents the most important key cytokine for MM cell regulation [4–6, 18]. MM cells proliferate in response to IL-6 under a paracrine-regulated manner and proliferate in close contact with IL-6-producing BMSCs [4, 5, 39]. On the other hand, myeloma cells are also known to produce their own IL-6 under an autocrine manner [40]. IL-6 secreted from BMSCs binds to IL-6 receptor (IL-6R) to initiate IL-6 signalling. IL-6R, which is generated in MM cells, binds to signal transducer membrane protein (gp130), which then stimulates the Janus kinases/signal transducer-activator of transcription (JAK/STAT) and GTPase/mitogen-activated protein kinase (Ras/MAPK) pathways. Both JAK/STAT and Ras/MAPK pathways play an important role in MM proliferation and inhibition of apoptosis [7]. Since IL-6 is a potent proliferative factor in MM cells, the suppression of IL-6 by CAM [2, 3, 22, 25, 28, 29, 30, 33, 34, 41–45] (Table 1) will lead to the inhibition on the growth of MM cells.

### IL-8 (CXCL8)

IL-8, a member of CXC chemokine family, was originally described as a neutrophil chemoattractant. IL-8 is known to promote cancer cell growth, survival, angiogenesis and metastasis in various human cancers. Herrero* et al* [46] demonstrated that IL-8 did not affect the growth of MM cells but protected them from death induced by serum starvation. Kline *et al* [47] reported that MM cells expressed IL-8 receptors (i.e., CXCR1 and CXCR2), BMSCs produced IL-8 in active MM and IL-8 production by BMSCs paralleled the MM disease activity. Pellegrino *et al* [48] demonstrated that IL-8 (CXCL8) and stromal cell-derived factor 1α (SDF-1α, a member of CXC chemokine which is also known as CXCL12) stimulate the proliferation and chemotaxis of human myeloma cells. Kikuchi *et al* [49] reported that CAM suppressed lipopolysaccharide (LPS)-induced IL-8 production by human monocytes through activator protein-1 (AP-1) and nuclear factor-κB (NF-κB) transcription factors. Thus, the suppression of IL-8 by CAM [2, 22, 25, 28, 30, 49, 50] (Table 1) might be related to MM cell reduction.

### IL-10

IL-10 is a cytokine with multiple and pleiotropic effects in immunoregulation and inflammation. IL-10 belongs to MGFs and represents a potent IL-6-unrelated MM cell proliferation factor but not a differentiation factor [51]. Gu *et al* [52] reported that IL-10 induced MM cell proliferation through an OSM autocrine loop. However, the studies on the influence of CAM on IL-10 showed conflicting results. Some investigators reported that CAM suppressed IL-10 production, secretion or regulation [26, 28, 44, 54, 55] (Table 1), whereas others [23, 29] (Table 1) showed the enhancement of IL-10 by CAM. These conflicting results might be attributed to different experimental conditions. Thus, it is very difficult to draw a definite conclusion on the effect of CAM on IL-10, pending further studies.

### IL-12

IL-12 is a cytokine that exerts potent antitumor activity through a combination of immunomodulatory and antiangiogenic mechanism. According to Airoldi *et al* [56], IL-12 mediates the development and maintenance of Th1 cells and induces IFN-γ production by Th1 and NK cells. They demonstrated that IL-12Rβ2 was expressed in primary MM cells but downregulated as compared with normal polyclonal plasmablastic cells and plasma cells. They showed that IL-12 reduced the proangiogenic activity of primary MM cells *in vitro* and significantly decreased the tumourgenicity of the NCI-H929 MM cells transplanted in SCID/NOD mice by inhibiting cell proliferation and angiogenesis. However, the study of CAM effect on IL-12 seems to show conflicting results. Some investigators reported that CAM enhanced IL-12 production or gene expression [44, 53–55], whereas others reported the suppressive effect of CAM on IL-12 [29, 30, 38] (Table 1). The contradictory results might be caused by the difference in the experimental settings. Further detailed studies are needed to define the actual effect of CAM on IL-12.

### IL-17

IL-17, a member of MGFs, is a proinflammatory cytokine produced by Th-17 cells. It is closely related to TGF-β, nerve growth factor, bone morphogenic protein and platelet-derived growth factor (PDGF) with similar structural motifs. IL-17 induces the expression of a number of chemokines and cytokines including IL-6, TGF-β, GCSF, GM-CSF, MMP and intercellular adhesion molecule-1 in a variety of cell types, including BMSCs [57]. Prabhala *et al* [57] demonstrated that Th17 cells and serum IL-17 increased in MM, and IL-17 promoted MM cell growth both *in vitro* and *vivo* through IL-17 receptors and inhibited Th1 responses [57]. The suppressive effect of CAM on IL-17 [30, 58] (Table 1) might be manifested as a reduction of MM cells.

### TNF-α

TNF-α, a member of TNF superfamily, is a major proinflammatory cytokine involved in the innate immune response. TNF-α is known to be produced mainly by activated macrophages. According to Jourdan *et al* [59], TNF-α is a survival and proliferation factor for human MM cells, though less potent than IL-6. Its survival activity is not affected by anti-IL-6 or anti-gp130 monoclonal antibodies, and it induces MM cells into the cell cycle to promote long-term MM cell growth. Hideshima *et al* [60] investigated the role of TNF-α using human IM-9 MM cells and demonstrated that TNF-α secreted from MM cells induced only a modest increase of cell proliferation, as well as MAPK and NF-κB activation. They also showed that TNF-α upregulated MAPK and NF-κB and markedly increased IL-6 secretion from BMSCs. Furthermore, they demonstrated that TNF-α induced the adhesion molecules on both MM cells (VLA-4, LFA-1 and MUC-1) and BMSCs (ICAM-1 and VCAM-1), which resulted in increased adhesion between MM cells and BMSCs. An increase in adhesion activity, in turn, induces IL-6 secretion from BMSCs, leading to MM cell proliferation. Therefore, the important role of TNF-α in MM is to augment paracrine IL-6-mediated MM cell growth. Thus, the suppression of TNF-α by CAM [23, 26–29, 33, 38, 41, 42, 61] (Table 1) might cause MM cell reduction.

### IFN-γ

IFN-γ is crucial for immunity against intracellular pathogens and tumour control. Cells from the innate immune system (e.g., NK cells, NKT cells, macrophages and myelomonocytic cells) and those from the adaptive immune system (e.g., Th1 cells, cytotoxic T lymphocytes and B cells) are known to produce IFN-γ [62, 63]. Antitumour potential of INF-γ is well known. Portier *et al* [64] experimentally demonstrated that IFN-γ inhibited IL-6-dependent MM cell growth and downregulated IL-6R. According to Klein and Bataille [4], IFN-γ downregulates the expression of IL-6R on MM cells and completely inhibits the IL-6-mediated MM cell proliferation. In this respect, some investigators reported that CAM suppressed IFN-γ production or gene expression [29, 33], whereas others [36, 44, 54, 55] (Table 1) presented conflicting results. The conflicting results might have arisen by the use of different materials and methods. Thus, we still cannot draw a definite conclusion about the effect of CAM on IFN-γ.

### VEGF/PDGF family

The VEGF/PDGF family belongs to MGFs. VEGF is a multifunctional cytokine that plays an important role in triggering tumour cell migration and angiogenesis. It also possesses a modest proliferative effect on myeloma cells. The production of VEGF in the bone marrow environment is upregulated by myeloma cell adhesion to BMSCs and by IL-6. Moreover, VEGF reciprocally enhances the secretion of IL-6 from BMSCs, suggesting the existence of paracrine interaction between MM cells and BMSCs [65]. PDGF plays a significant role in blood vessel formation, mitogenesis and chemotaxis. PDGF is produced mainly by megakaryocytes and alpha granules of platelets and is released by platelets on activation. It is also produced by other cells such as smooth muscle cells, activated macrophages and endothelial cells. It was shown that PDGF receptor (PDGFR)-α and β were frequently expressed on MM cells of newly diagnosed MM patients, and high PDGFR-β expression at diagnosis was associated with advanced stage of the disease [66]. PDGF-β/PDGFR-β kinase axis plays an important role in angiogenesis and proliferation in MM. The suppressive effect of CAM on VEGF/PDGF [29, 30, 67] (Table 1) might thus be led to MM cell reduction.

### TGF-β

TGF-β, a member of MGFs, is a multifunctional cytokine belonging to the TGF superfamily and has pleiotropic biological effect in haematopoiesis and tumourigenesis. It is produced by a large variety of cells including epithelial cells, fibroblasts, eosinophils, macrophages and Treg cell [62]. Urashima* et al* [68] reported that MM cells secreted more TGF-β1 than splenic B-cells or CD40L pretreated B-cells, and BMSCs of MM produced more TGF-β1 than normal BMSCs [53]. According to Dong and Blobe [69], TGF-β is secreted at higher levels from both MM cells and BMSCs in MM and the increased production of TGF-β correlates well with increased IL-6 and VEGF secretion by BMSCs. Furthermore, neutralising antibody to TGF-β can block increased IL-6 and VEGF secretion, supporting TGF-β as the major inducer for the secretion of IL-6 and VEGF from BMSCs. The suppression of TGF-β by CAM [25, 43, 61] (Table 1) might cause MM cell reduction.

### FGF2 (Basic FGF)

FGF2 (also called basic FGF) is a growth factor with angiogenetic properties and represents one of the MGFs [6]. FGF2 is known to be a significant mediator supporting MM cell expansion and survival. According to Mahtouk *et al* [6], BMSCs are the main source of FGF2, and they demonstrated that FGF2 gene was expressed in MM cells, but its expression was lower than that of BMSCs. The suppression of FGF2 by CAM [29] (Table 1) was noted.

### G-CSF

G-CSF, a member of MGFs, is a potent growth factor for MM cells as well as a hematopoietic growth factor with structural homology to IL-6. G-CSF receptor shares some homology with gp130. Both G-CSF and IL-6 induce the activation of nuclear factor for IL-6 (NF-IL-6), which is a transcription factor involved in the synthesis of IL-6 [18]. The suppressive effect of CAM on G-CSF [22] (Table 1) was documented.

### GM-CSF

GM-CSF, a member of MGFs, is a hematopoietic growth factor which has a broad impact on haematopoiesis; it stimulates the development and differentiation of committed stem cells to neutrophils, eosinophils and monocytes. According to Zhang *et al* [17], GM-CSF is a strong stimulator of* in vitro* myeloma cell proliferation by potentiating the response of myeloma cells to IL-6. The suppression of GM-CSF by CAM [22, 29] (Table 1) might indirectly lead to MM cell reduction.

### Matrix metalloproteinases (MMPs)

MMPs play an important role in cell growth, invasion, angiogenesis, metastasis and bone degradation, which are all important events in the pathogenesis of cancer. Van Valckenborgh *et al* [70] showed the multifunctional role of several MMPs in the development of MM, especially MMP-9, which is closely related to angiogenesis. The suppressive effect of CAM on MMP-9 [29, 30, 61, 71] (Table 1) might be related to MM cell reduction through the downregulation of angiogenesis.

Taken together, CAM can suppress MMP-9 and the following MGFs: IL-1, IL-2, IL-5, IL-6, IL-8, IL-17, TNF-α, PDGF, VEGF, TGF-β, FGF2, G-CSF and GM-CSF (Table 1). Of these MGFs, IL-6 seems to be a crucial factor for MM cell proliferation. Thus, it is probable that the suppression of these MGFs by CAM will cause MM cell reduction. On the contrary, CAM enhanced IL-4 activity [36] (Table 1). However, IL-10, IL-12 and IFN-γ, which are positively or negatively involved in MM cell proliferation, were reported to show the conflicting patterns of response to CAM (Table 1). Thus, it is difficult to draw a definite conclusion on the actual effect of CAM on these cytokines. The effects of CAM on other MGFs (IL-16, IL-22, IL-23, IGF1, HGF, MIP**α**, GDF-15, PTN, BDNF, CNTF, OSM, LIF, CCLF1, BAFF, APRIL, AREG, NRG, HB-EGF, Wnt family members, Jagged family members and other suppressive cytokines against MM [e.g., IL-27]) have not yet been investigated.

## CAM-monotherapy for MM

The first report on the use of CAM in MM treatment dates back to 1997. Durie *et al* [72] pioneered a unique trial of CAM monotherapy to treat active MM. In their trial, 500 mg of CAM was administered to the patients twice a day approximately for 1 year as the longest follow-up period. They reported that greater than 50% response rate was obtained. However, several responding patients in their study were on concurrent antimyeloma agents that include steroids. Moreau *et al* [73] reported that no response was achieved in 35 MM patients receiving CAM, at 500 mg, twice a day for 4–20 weeks (median 8 weeks) without chemotherapy or steroids; MM progressed in 80% of cases, requiring other therapeutic approaches. Musto* et al* [74] reported that among 38 evaluable patients with relapsed or refractory MM (RRMM), who were treated with CAM, at 500 mg, twice a day for 12 weeks, an objective response or minor response (MR) was obtained only in one and two patients, respectively. On the other hand, Stewart *et al* [75] reported no significant response among 20 evaluable MM patients at various phases of the disease treated with CAM alone. CAM monotherapy seems to have no effect on treating MM.

## Low-dose thalidomide and low-dose Dex combined with CAM (Biaxin^®^) (BLTd) for MM treatment

On contrary to the low efficacy of CAM-monotherapy, CAM has added value when combined with thalidomide or its analogues and Dex. Coleman *et al* [76] pioneered low-dose thalidomide and low-dose Dex combined with CAM (BLTd) to treat previously untreated or treated MM as well as Waldenström’s macroglobulinemia and succeeded in achieving a high overall response rate (ORR) of 93%. Niesvizky *et al* [77] carried out a prospective, randomised trial which compared the safety and efficacy of standard pulsed Dex to those of low-dose thalidomide and low-dose Dex (LTd). A total of 23 advanced MM patients were randomised to either pulsed D or LTd. The patients given pulsed Dex displayed almost similar drops in paraprotein at 8 weeks of treatment as the LTd patients (median: 45% versus 52%). Nine patients (D=3, LTd=6) showed a <50% response (median 26%, SD 15.9) to induction therapy after 8 weeks. Then, Niesvizky *et al* [77] added CAM to the pulsed Dex or LTd treatments, and they showed that the addition of CAM produced further tumour mass reduction at 16 and 20 weeks of treatment. Overall response rate (>50% response in paraprotein) for pulsed Dex and LTd patients was 80%. They suggested that the addition of CAM contributes to tumour mass reduction by potentiating Dex’s effect. Morris* et al* [45] also reported ORR of 96% in RRMM patients when they received BLTd.

## Lenalidomide (Len: Revlimid^®^) and Dex/low-dose Dex combined with CAM (BiRD/BiRd) or pomalidomide (Pom) and low-dose Dex combined with CAM (Clapd) for MM treatments

Len is one of the immunomodulatory drugs (IMiDs) with a structural and functional analogue of thalidomide [78, 79]. In 2008, Niesvizky *et al* [80] first reported the BiRD regimen for the treatment of naive symptomatic MM. The addition of CAM allowed a significant reduction of Dex, and an objective response rate of 90.3% was achieved. A combined stringent and conventional CR rate of 38.9% was achieved, and 73.6% of the patients achieved at least a 90% decrease in M-protein levels. Gay* et al* [81] performed a case-matched study and compared the efficacy and toxicity of BiRd versus Len combined with low-dose Dex (Rd) for newly diagnosed MM. BiRd regimen, compared to Rd regimen, showed increased CR rates (45.8% versus 13.8%), very good partial response (VGPR) rates (73.6% versus 33.3%) and a longer progression-free survival (PFS) (median: 48.3 months versus 27.5 months). The superiority of BiRd regimen was thus suggested. Rossi *et al* [82] reported that 72 previously untreated MM patients who were treated with BiRd achieved ORR of 93% (VGPR or better of 68%), and median progression-free survival (PFS) was 49 months. They demonstrated that BiRd was highly effective.

Pomalidomide (Pom) is a distinct immunomodulatory agent similar to Len. Mark *et al* [83] reported that Clapd proved to be effective for treating relapsed or refractory MM (RRMM). In their study, 117 patients with heavily pretreated RRMM including prior Len-treated RRMM were analysed. ORR (≧PR) and clinical benefit response rates (≧MR) were 60% and 67%, respectively. They concluded that Clapd is a highly effective and tolerable regimen for heavily treated RRMM.

## Steroid-enhancing/sparing effect of CAM

The role of CAM in BiRd, BLTd and Clapd regimen has been attributed to its steroid-enhancing/sparing effect [3, 76, 77, 80, 81, 82, 84]. It is well known that CAM is a potent CYP3A4 inhibitor [3, 84]. CAM added in BiRd, BLTD and Clapd regimen slows the hepatic clearance of Dex, leading to the augmentation of Dex responsiveness to MM cells [3]. Fost *et al* [85] investigated the inhibitory effect of CAM on the elimination of methyl-prednisolone (methyl-PSL) and prednisolone (PSL) in six patients with asthma. In their study, the patients received a single oral dose of methyl-PSL (40 mg/1.73 m^2^) or PSL (40 mg/1.73 m^2^) before and post administration of 7 days of CAM (500 mg twice a day). Pre- and post-CAM pharmacokinetic profiles of the two steroids showed a 65% reduction in clearance of methyl-PSL but no significant reduction in clearance of PSL. The interactions of CAM with methyl-PSL and PSL were found to be different. To the best of the authors’ knowledge, studies directly evaluating the effect of CAM on Dex metabolism have not been reported.

## Reversibility of drug resistance by CAM

Juge-Morineau *et al* [86] demonstrated that the gp130 family cytokines, such as IL-6, LIF and OSM, inhibit the antiproliferative effect of Dex on human MM cells. Yang and Lin [87] reviewed the mechanisms of resistance against Dex in MM. According to them, IL-6 induces drug resistance against Dex by stimulating JAK/STAT to BCL-XL/MCL1 transduction cascade and PI3K/AKT transduction cascade, consequently leading to the upregulation of antiapoptotic signalling [88, 89, 90]. It is noteworthy that IGF-1, a member of MGFs, is also involved in the development of drug resistance against current standard-of-care agents for MM [91]. MM cells express IGF-I receptor, and its stimulation activates two distinct signal transduction cascades (PI3K/Akt and MEK/ERK cascades), leading to the proliferation of MM cells as well as protection against apoptosis [91, 92]. As far as we know, no studies demonstrating the effect of CAM on IGF-1 have been done. Since CAM can potently suppress IL-6, the reversibility of drug resistance including steroid resistance by CAM in MM treatments seems more important than the CYP3A4-related steroid-sparing/enhancing effect thus far reported.

## Specific character of MM as a monoclonal immunoglobulin (Ig)-producing neoplasm

**Figure 1.**
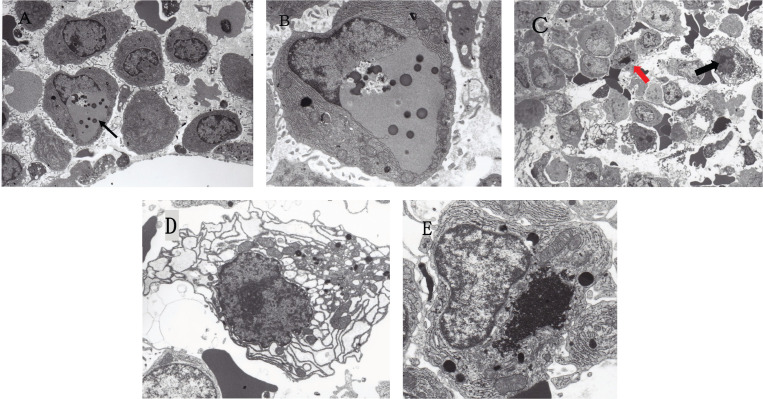
Electron micrographs of MM cells reflecting ER stress. (A) Low power view of the bone marrow (IgA-κ type MM) obtained before the initiation of chemotherapy. An arrow indicates a myeloma cell with an autophagosome. Original magnification ×2,000. (B) Enlarged view of a myeloma cell indicated by an arrow in (A) showing a well-developed autophagosome formed in perinuclear space. Several dense bodies (lysosomes) are scattered in the autophagosome. Original magnification ×5,000. (C) Low power view of the bone marrow (Bence Jones-λ type MM) obtained before the initiation of chemotherapy. A black arrow indicates a myeloma cell showing dilated RER, and a red arrow indicates a myeloma cell with aggresome. Original magnification ×1,000. (D) Enlarged view of a myeloma cell indicated by a black arrow in (C) showing remarkably dilated RER in the cytoplasm, suggesting RER-stress. Original magnification ×4,000. (E) Enlarged view of a myeloma cell indicated by a red arrow in (C) showing the well-developed aggresome formation in the cytoplasm. Original magnification ×4,000.

Since MM is characterised by the uncontrolled cell growth of monoclonal immunoglobulin-producing neoplastic plasma cells, the large quantities of unfolded or misfolded Ig production itself trigger endoplasmic reticulum stress (ER stress) which is followed by the unfolded protein response (UPR) [93, 94, 95, 96]. According to Obeng *et al* [95], MM cells are inherently sensitive to proteasome inhibitors because of their large volume of Ig production which requires the expression of physiologic UPR genes. If the ER stress is severe or prolonged, UPR activation leads to cell-cyclic arrest and the induction of apoptosis of MM cells. Thus, it is probable that MM is a specific neoplasm susceptible to proteasome-, autophagy- and histone deacetylase 6 (HDAC6)-inhibitors. In this study, the bone marrow of MM patients obtained before chemotherapy showed that some of the myeloma cells were already under ER stress at the electron microscopic level (Figure 1).

## Role of CAM as a potent autophagy inhibitor

It is well known that CAM is a potent and continuous inhibitor of autophagy. Nakamura *et al* [97] analysed the direct effect of CAM on MM cells* in vitro*. They demonstrated that CAM attenuated autophagy by blocking the late phase of the autophagic process, probably after the fusion of autophagosomes with lysosomes at clinically relevant concentrations (6–50 μg/mL). In other words, CAM halts the autophagy process and induces the inhibition of MM cell growth. Thus, CAM can serve as a potential adjuvant for MM treatment modalities, where autophagy is used by the tumour as an escape mechanism from apoptosis.

## Synergistic effect of CAM with proteasome inhibitor or histone deacetylase 6 (HDAC6) inhibitor

**Figure 2.**
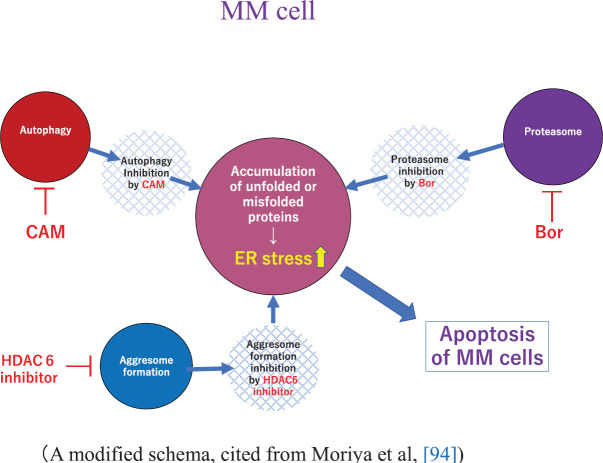
A proposed schema showing apoptosis of MM cells by autophagy, proteasome and aggresome-inhibitors. A modified schema, cited from Moriya et al, [94]. Abbreviations: Bor, bortezomib; CAM, clarithromycin; ER, endoplasmic reticulum; HDAC6, histone deacetylase 6; MM, multiple myeloma.

Bortezomib (Bor, Velcade^®^), the first-in-class proteasome inhibitor of the 26S proteasome, was initially approved for the treatment of patients with RRMM as a single agent [98]. Bor is now widely used in combination regimens: Bor, thalidomide and low-dose Dex (VTd) [99], Bor and low-dose Dex (Vd) [100], Bor, Len and low-dose Dex (VRd) [101], Bor, cyclophosphamide and low dose Dex (VCd) [102], Bor, melphalan and prednisolone (VMP) [103], daratumumab, Bor and low-dose Dex (DVd) [104] or pomalidomide, Bor and low-dose Dex (PVd) [105] in RRMM and newly diagnosed MM. The effect of Bor was reported due to its blocking of the ubiquitin-proteasome system, leading to the accumulation of unfolded or misfolded protein in the ER in myeloma cells. This results in ER stress followed by a coordinated cellular response known as UPR [93, 94, 95]. UPR is known to induce activation of the chaperone protein GRP-78 (Bip: binding immunoglobulin protein) to maintain ER integrity, and it also upregulates the transcription factor CHOP (i.e., C/EBP homologous protein) to mediate cell death when ER stress is beyond the tolerance of the cell adaptation [95]. The combined inhibition of the ubiquitin (Ub)-proteasome system by Bor and autophagy-lysosome system by CAM synergistically activates UPR, resulting in MM cell apoptosis. Moriya* et al* [93] experimentally demonstrated that combined treatment of CAM and Bor resulted in increased cytotoxicity as compared to the use of Bor alone. The effectiveness of this combined treatment with CAM, Len and Bor was recently demonstrated at the clinical level [106]. The apoptosis-inducing effect was further enhanced when vorinostat (HDAC6 inhibitor), which is known to inhibit aggresome formation, was added to Bor and CAM [94] (Figure 2). However, no clinical studies have been done using this combination.

Carfilzomib (Kyprolis^®^) is a selective and irreversible epoxyketone proteasome inhibitor of the 20S proteasome. Recently, carfilzomib, Len and low-dose Dex (KRd) [107] combined with CAM (KRd-CAM) were also found to be effective for treating refractory MM (unpublished data).

## Safety of long-term administration of CAM

Antibacterial treatment with CAM is usually recommended for a duration of 1–2 weeks. Safety of the long-term administration of CAM has been proved [2, 108]. However, there remains a concern regarding the development of resistance of *Streptococcus pneumoniae* against CAM due to the long-term administration of CAM. Recently, concerns have been raised on the long-term effect regarding the risk of cardiovascular events and mortality in both patients with stable coronary heart disease and patients without heart disease, following the short-term use of CAM (e.g., daily for 2 weeks) [3].

## Adverse effects of CAM

The frequent and common adverse effects of CAM are diarrhoea, nausea, abnormal taste, dyspepsia, abdominal pain/discomfort, headache, insomnia, tooth discolouration, smell loss and taste loss. These adverse reactions are usually mild in intensity and resolve after the discontinuation of treatment. All other adverse reactions are uncommon or rare [3]. However, in post-marketing surveillance, allergic reactions ranging from urticaria and mild skin eruptions to rare cases of anaphylaxis, Stevens-Johnson syndrome, toxic epidermal necrosis, liver dysfunction, somnolence and confusion have occurred [https://www.accessdata.fda.gov/drugsatfda_docs/label/2012/050662s044s050,50698s026s030,050775s015s019lbl.pdf]. In particular, CAM is also known to prolong QT interval. Rarely, it can be fatal, leading to fatal ventricular arrhythmias, including ventricular tachycardia and torsades de pointes [109]. Thus, CAM therapy should not be given to the patients with a history of QT prolongation and arrhythmia. Since CAM inhibits CYP3A4, the coadministration of CAM with some drugs metabolised by CYP3A4 should be carefully done.

## Indirect effects of CAM against virus infections

Recently, the World Health Organisation (WHO) has declared the new coronavirus (SARS-CoV-2) outbreak a pandemic. Several repurposing drugs (e.g., remdesivir, favipiravir, lopinavir·ritonavir, chloroquine, nafamostat, ciclesonide, tocilizumab and ivermectin) possibly effective for treating the disease (COVID-19) caused by SARS-CoV-2 have been tested. However, no definite drugs for treating COVID-19 have yet been established after the declaration by the WHO of the pandemic. Recently, the FDA of the United States of America authorised the emergency use of remdesivir for treating COVID-19.

In general, antibiotics are known to be ineffective against virus infection. Shinahara *et al* [110] treated 195 influenza-A-infected children with oseltamivir (OSV) and zanamivir (ZNV) with or without CAM. They demonstrated that the combination of CAM plus OSV or ZNV boosted and restored the production of mucosal secretory IgA (S-IgA) and systemic IgG. Furthermore, they reported that CAM supplementation reduced the reinfection rate in the subsequent year in the patients treated with OSV and ZNV. They suggested the possibility that CAM enhanced influenza virus-specific S-IgA production through the induction of IgA class switching recombination.

It is interesting to note that hydroxychloroquine treatment combined with azithromycin, 15-membered macrolide, reinforced viral load reduction/disappearance in COVID-19 patients [111]. Just recently, Millán-Oñate *et al* [112] first reported that a 34-year-old Columbian man with COVID-19 pneumonia was successfully recovered after receiving chloroquine and CAM. Emerging data suggest that many patients with COVID-19 may die due to an excessive response of their immune system, characterised by the abnormal release of circulating cytokines including IL-1β, IL-6, IL-12, IL-18, TNF-α, TGF-β, GM-CSF, IFN-γ and various chemokines. This phenomenon is referred to as cytokine release syndrome (CRS), and a crucial role seems to be played by IL-6. CRS plays a major role in the deterioration of COVID-19 patients with pneumonia to acute respiratory distress syndrome, cumulating in systemic inflammation and multiorgan failure [113]. In addition, clarithromycin is also known to be effective for treating organising pneumonia [114]. Cai *et al* [28] demonstrated that spontaneous and lipopolysaccharide-stimulated alveolar macrophages from patients with BOOP upregulated the production of TNF-α, soluble TNF receptor 1/2, IL-1β, IL-6, IL-8, IL-10 and chemokine ligand 18. They suggested that CAM significantly attenuated or inhibited the production of these cytokines. Considering the pleiotropic immunomodulative effects of CAM, the suppressive effects of CAM on cytokines related to CRS, particularly on IL-6, should be noted. The administration of CAM as a single agent or combined with other drugs might be worth a try for treating COVID-19. One trial of CAM against COVID-19 has already started in Greece in May, 2020 (https://www.clinicaltrialsregister.eu/ctr-search/trial/2020-001882-36/GR).

## Conclusion

We reviewed the current preclinical, clinical and experimental evidence for supporting the efficacy of CAM for treating MM, especially when it is combined with IMiDs, steroids or proteasome inhibitors. IMiDs, steroids, proteasome inhibitors and CAM might synergistically play an important role in inducing apoptosis of MM cells. In addition, the reversibility of drug resistance by CAM to chemotherapeutic drugs including steroids seems to be more important than steroid-sparing/enhancing effect thus far reported in MM treatments. Since multiple myeloma is characterised by the uncontrolled cell growth of monoclonal Ig-producing neoplastic plasma cells, the large quantities of unfolded or misfolded immunoglobulin production itself may trigger ER stress. Thus, MM is a specific neoplasm particularly susceptible to proteasome, autophagy and HDAC6 inhibitors. The development of CAM analogues specific for more potent immunomodulatory effects may provide a novel approach to MM treatment in the future.

In addition, considering the pleiotropic effects of CAM on cytokines including IL-6 and its indirect antiviral effects, CAM might be worth a try for treating COVID-19.

## Abbreviations

AP-1: activator protein
APRIL: a proliferation-inducing ligand
AREG: amphiregulin
BAFF: B-cell activating factor
bFGF: basic fibroblast growth factor
EGF: epidermal growth factor
BCL-xL: B-cell lymphoma-extralarge
Bip: chaperone protein GRP-78
BiRd: Len and low-dose Dex combined with CAM
BDNF: brain-derived neurotrophic factor
BMSCs: bone marrow stromal cells
BLTd: low-dose thalidomide and low-dose DE combined with CAM
BOOP: bronchiolitis obliterans organising pneumonia
Boor: bortezomib
CAM: clarithromycin
CCLF1: cardiotrophin-like cytokine factor 1
CHOP: C/EBP homologous protein
Clapd: Pom and low-dose Dex combined with CAM
CNTF: ciliary neurotrophic factor
CR: complete response
Dex: dexamethasone
ER: endoplasmic reticulum
G-CSF: granulocyte colony-stimulating factor
GDF15: growth/differentiation factor15
GM-CSF: granulocyte macrophage colony-stimulating factor
gp130: glycoprotein 130
HB-EGF: heparin-binding EGF-like growth factor
HDAC6: histone deacetylase 6
HGF: hepatocyte growth factor
Ig: immunoglobulin
IGF-1: insulinlike growth factor-1
ICAM-1: intercellular adhesion molecule-1
IL: interleukin
IL-6R: IL-6-receptor
IMiDs: immunomodulatory drugs
IFN-γ: interferon-γ
JAK/STAT: Janus kinases/signal transducer-activator of transcription
KRd: Kyprolis^®^, Len and low-dose Dex
Len: lenalidomide
LFA-1: lymphocyte function-associated antigen-1
LIF: leukaemia inhibitory factor
LTD: Len, low-dose thalidomide and Dex
MCL-1: myeloid cell leukaemia sequence-1
MGF: myeloma growth factor
MIPα: macrophage inflammatory protein α
MM: multiple myeloma
MMP: matrix metalloproteinase
MR: minor response
MUC-1: muchin-1
NF-IL-6: nuclear factor for IL-6
NF-κB: nuclear factor-κB
NRG1~4: neureglin1~4
NK: natural killer
NKT: natural killer T
ORR: overall response rate
OSM: oncostatin M
PFS: progression-free survival
PI3K/AKT: phosphatidylinositol-3 kinase/AKT kinase
PDGF: platelet-derived growth factor
Pom: pomalidomide
PR: partial response
PSL: prednisolone
PTN: pleiotrophin
Ras/MAPK: GTPase/mitogen-activated protein kinase
Rd: Len combined with low-dose dex
RRMM: relapsed or refractory multiple myeloma
SDF-1: stromal cell-derived factor 1
S-IgA: secretory immunoglobulin A
TGF-β: transforming growth factor-β
TNF-α: tumour necrosis factor-α
UPR: unfolded protein response
VEGF: vascular endothelial growth factor
VCAM-1: vascular cell adhesion molecule-1
VGPR: very good partial response
VLA: very late activating antigen
Wnt5A/10B/16: Wnt family members

## Competing interests

All of the authors declare that they have no conflicts of interest.

## Funding

This study was not supported by any grant.

## Figures and Tables

**Table 1.** Cytokines and enzyme, which are positively or negatively influenced by CAM, with respect to MM cell proliferation.

Cytokines suppressed by CAM		
Cytokines	References	Supplementary explanations
**IL-1α/β**	Takeshita *et al* [21]	Murine peritoneal macrophages precultured with CAM showed diminished IL-1 production.
	Matsuoka* et al* [22]	An inhibitory effect of CAM on costimulatory molecule expression and cytokine production by cultured synoviocytes was investigated. CAM suppressed the production of IL-1β in human synoviocytes obtained from rheumatoid arthritis (RA) or osteoarthritis (OA) patients.
	Morikawa *et al* [23]	CAM suppressed the synthesis of IL-1α/β by lipopolysaccharide (LPS)-stimulated healthy human monocytes.
	Miyanohara *et al* [24]	CAM suppressed IL-1β gene expression in human nasal epithelial cell.
	Čulić *et al* [25]	Anti-inflammatory effects of macrolide antibiotics (review of literature).
	Fonseca-Aten* et al* [26]	Children with a history of recurrent wheezing or asthma, who presented with an acute exacerbation of wheezing, were treated with CAM. Nasopharyngeal concentrations of IL-1β of the patients decreased after CAM treatment.
	Perić *et al* [27]	Nasal polyp patients (nonallergic and allergic) were treated with CAM for 8 weeks. Following CAM treatment, IL-1β in nasal fluid of allergic patients significantly decreased.
	Cai *et al* [28]	CAM inhibited spontaneous and LPS-stimulated IL-1β production by alveolar macrophages in bronchiolitis obliterans organising pneumonia (BOOP).
	Zeng* et al* [29]	Anti-inflammatory effect of CAM on ethmoid mucosal tissue of chronic rhinosinusitis (CRS) was investigated. CAM suppressed the protein production of IL-1β in sinonasal mucosa of CRS without nasal polyp.
	Zimmermann *et al* [30]	The immunomodulatory effects of macrolides (review of literature).
**IL-2**	Morikawa *et al* [32]	The immunomodulatory effects of three macrolides including CAM on human T-cell function were investigated. CAM suppressed IL-2 production induced by mitogen-stimulated T-cells.
	Morikawa *et al* [33]	The effects of macrolides including CAM on the generation of human Th1- and Th2-type cytokines were investigated. CAM showed a potent inhibitory activity on the release and gene expression of IL-2 induced by Con-A-stimulated human T-cells.
	Sugiyama *et al* [34]	Differing effects of macrolides including CAM on cytokine production by murine dendritic cells were investigated. CAM decreased IL-2 production by co-cultured dendritic cells and T-cells.
	Zimmermann *et al* [30]	The immunomodulatory effects of macrolides (review of literature).
**IL-5**	Kraft* et al* [38]	The asthma patients treated with CAM demonstrated a reduction of IL-5 mRNA in bronchoalveolar lavage (BAL).
	Morikawa *et al* [33]	The effect of macrolides including CAM on the generation of human Th1- and Th2-type cytokines was investigated. CAM showed an inhibitory activity on the release and gene expression of IL-5 induced by Con-A-stimulated human T-cells.
	Zeng *et al* [29]	Anti-inflammatory effect of CAM on ethmoid mucosal tissue of CRS was investigated. CAM suppressed the protein production of IL-5 in sinonasal mucosa of eosinophilic CRS with nasal polyp.
	Zimmermann *et al* [30]	The immunomodulatory effects of macrolides (review of literature).
**IL-6**	Sakamoto et al [41]	Patients with NSCLC were treated with CAM. The level of serum IL-6 was significantly depressed after CAM treatment.
	Matsuoka et al [22]	CAM significantly suppressed the production of IL-6 in IL-1β-added cultured synoviocytes obtained from RA and OA patients.
	Khan *et al* [42]	The effect of IL-6 production by LPS- and Pansorbin-stimulated human monocytes was investigated. CAM significantly decreased an IL-6 production.
	Sassa *et al* [43]	The therapeutic effect of CAM on subcutaneously inoculated mammary adenocarcinoma cells in rats was investigated. The expression of IL-6 gene of spleen cells of tumour-bearing rats significantly decreased after CAM treatment.
	Majima* et al* [44]	Mice were inoculated subcutaneously with Lewis lung carcinoma (LLC) cells. The treatment of CAM (for 7 days) started 1 week after tumour inoculation. Immediately after finishing the treatment, the mice were sacrificed, and the expression of IL-6 mRNA in spleen cells was evaluated. The expression of IL-6 mRNA decreased after CAM treatment.
	Čulić *et al* [25]	Anti-inflammatory effects of macrolide antibiotics (review of literature).
	Morikawa *et al* [33]	The effect of macrolides including CAM on the generation of human Th1- and Th2-type cytokines was investigated. CAM showed an inhibitory activity on the release and gene expression of IL-6 induced by Con-A stimulated human T-cells.
	Sugiyama *et al* [34]	The differing effects of macrolides including CAM on cytokine production by murine dendritic cells were studied. CAM decreased IL-6 production in co-cultured dendritic cells and T-cells.
	Morris* et al* [45]	Phase II study to treat patients with relapsed and refractory MM. With respect to the effect of added CAM on MM, the possibility that CAM acts through suppression of production of various cytokines including IL-6 was suggested.
	Kanoh and Rubin [2]	The mechanisms of action and clinical application of macrolides as immunomodulatory medications (review of literature).
	Cai *et al* [28]	CAM inhibited spontaneous and LPS-stimulated IL-6 production by alveolar macrophages in BOOP.
	Van Nuffel *et al* [3]	Repurposing drugs in oncology (ReDO)-CAM as an anticancer agent (review of literature).
	Zeng *et al* [29]	Anti-inflammatory effect of CAM on ethmoid mucosal tissue of CRS was investigated. CAM suppressed the protein production of IL-6 in sinonasal mucosa of CRS.
	Zimmermann *et al* [30]	The immunomodulatory effects of macrolides (review of literature).
**IL-8**	Matsuoka *et al* [22]	CAM suppressed the production of IL-8 by IL-1β-added human synoviocytes obtained from RA or OA patients.
	Suzuki *et al* [50]	The effect of CAM on IL-8 secretion from cultured human nasal epithelial cells was studied. The LPS-stimulated IL-8 secretion was significantly inhibited by CAM.
	Čulić *et al* [25]	Anti-inflammatory effects of macrolide antibiotics (review of literature).
	Kikuchi *et al* [49]	The effects of CAM on IL-8 production using stimulated human peripheral monocytes and human monocytic leukaemia line, THP-1, were investigated. CAM suppressed the production of IL-8 induced by stimulated monocytes and THP-1 cells.
	Kanoh and Rubin [2]	The mechanisms of action and clinical application of macrolides as immunomodulatory medications (review of literature).
	Cai *et al* [28]	CAM induced a dose-dependent attenuation of LPS-stimulated IL-8 production by alveolar macrophages in BOOP.
	Zimmermann *et al* [30]	The immunomodulatory effects of macrolides (review of literature).
IL-10*	Majima et al [54]	Patients with unresectable NSCLC were treated with CAM for 3 months. The expression of IL-10 mRNA was measured during CAM treatment. The expression of IL-10 mRNA significantly decreased during CAM treatment.
	Majima et al [44]	Patients with advanced NSCLC were treated with CAM. The expression of IL-10 mRNA in PBMNCs was evaluated during CAM treatment. In an additional experimental model, the mice were inoculated subcutaneously with LLC cells and treated with CAM for 1 week. Thereafter, the mice were sacrificed, and the expression of IL-10 mRNA in spleen cells was evaluated. The expression of IL-10 mRNA in PBMNCs and the spleen cells decreased after CAM treatments.
	Majima *et al* [55]	Patients with advanced NSCLC were treated with CAM for 3 months. The expression of IL-10 mRNA in the patients’ PBMNCs decreased during CAM treatment.
	Fonseca-Aten* et al* [26]	Children with an acute exacerbation of recurrent wheezing were enrolled in double-blind, randomised trial of CAM. The nasopharyngeal concentrations of IL-10 were significantly decreased in children treated with CAM.
	Cai *et al* [28]	CAM inhibited the LPS-stimulated IL-10 production by alveolar macrophages in BOOP.
IL-12*	Kraft* et al* [38]	The asthma patients treated with CAM demonstrated a reduction of IL-12 mRNA in BAL.
	Zeng *et al* [29]	Anti-inflammatory effect of CAM on ethmoid mucosal tissue of CRS was investigated. CAM suppressed the protein production of IL-12 in sinonasal mucosa of CRS.
	Zimmermann *et al* [30]	The immunomodulatory effects of macrolides (review of literature).
**IL-17**	Fouka *et al* [58]	Patients with stable non-cystic fibrosis bronchiectasis were treated with CAM. IL-17 concentrations in exhaled breath condensate (EBC) and peripheral blood Th17 cells were evaluated. Post-treatment absolute count of CD4^ +^IL-17^+^ cells in PB and IL-17 levels in EBC decreased significantly.
	Zimmermann *et al* [30]	The immunomodulatory effects of macrolides (review of literature).
**TNF-α**	Sakamoto *et al* [41]	Patients with NSCLC were treated with CAM. The level of serum TNF-α significantly decreased after CAM treatment.
	Morikawa *et al* [23]	CAM suppressed the synthesis of TNF-α by LPS-stimulated healthy human monocytes.
	Khan *et al* [42]	The effect of IL-6 production by LPS- and Pansorbin-stimulated human monocytes was investigated. CAM significantly decreased TNF-α production.
	Sassa* et al* [61]	Rat mammary adenocarcinoma cells were treated with CAM, and total RNAs were extracted from the tumour. CAM inhibited the expression of TNF-α gene.
	Kraft* et al* [38]	Asthma patients treated with CAM demonstrated a reduction of TNF-α mRNA in BAL.
	Morikawa *et al* [33]	The effect of macrolides including CAM on the generation of Th1- and Th2-type cytokines by mitogen-stimulated human T-cells was investigated. CAM showed a potent inhibitory activity on the release and gene expression of TNF-α.
	Fonseca-Aten* et al* [26]	Children with an acute exacerbation of recurrent wheezing were treated with CAM. The nasopharyngeal concentrations of TNF-α significantly decreased after CAM treatment.
	Perić *et al* [27]	Patients with nasal polyp (nonallergic and allergic) received CAM treatment for 8 weeks. Following the treatment, TNF-α in nasal fluid of allergic patients significantly decreased.
	Cai *et al* [28]	CAM inhibited the LPS-stimulated TNF-α production by alveolar macrophages in BOOP.
	Zeng *et al* [29]	Anti-inflammatory effect of CAM on ethmoid mucosal tissue of CRS was investigated. CAM suppressed the protein production of TNF-α in sinonasal mucosa of CRS without nasal polyp and eosinophilic CRS with nasal polyp.
IFN-γ*	Morikawa et al [33]	Anti-inflammatory effect of CAM on ethmoid mucosal tissue of CRS was investigated. The effect of macrolides including CAM on the generation of Th1- and Th2-type cytokines by mitogen-stimulated human T-cells was investigated. CAM showed a potent inhibitory activity on the release and gene expression of IFN-γ.
	Zeng et al [29]	Anti-inflammatory effect of CAM on ethmoid mucosal tissue of CRS was investigated. CAM suppressed the protein production of IFN-γ in sinonasal mucosa of CRS.
**Growth factors and enzyme suppressed by CAM**		
**Cytokines enzyme**	**References**	**Supplementary explanations**
**PDGF**	Zeng *et al* [29]	Anti-inflammatory effect of CAM on ethmoid mucosal tissue of CRS was investigated. CAM suppressed the protein production of PDGF in sinonasal mucosa of CRS.
**VEGF**	Hu *et al* [67]	CAM decreased the protein expression of VEGF in the nasal mucosa of CRS with nasal polyp.
	Zeng *et al* [29]	Anti-inflammatory effect of CAM on ethmoid mucosal tissue of CRS was investigated. CAM suppressed the protein production of VEGF in sinonasal mucosa of CRS.
	Zimmermann *et al* [30]	The immunomodulatory effects of macrolides (review of literature).
**TGF-β**	Sassa *et al* [43]	The therapeutic effect of CAM on subcutaneously inoculated mammary adenocarcinoma cells in rats was investigated. The expression of TGF-β gene of spleen cells of tumour-bearing rats significantly decreased after CAM treatment.
	Sassa* et al* [61]	Rat mammary adenocarcinoma cells were treated with CAM, and total RNAs were extracted from the tumour. CAM inhibited the expression of TGF-β gene.
	Čulić *et al* [25]	Anti-inflammatory effects of macrolide antibiotics (review of literature).
**FGF2 (basic FGF)**	Zeng *et al* [29]	Anti-inflammatory effect of CAM on ethmoid mucosal tissue of CRS was investigated. CAM suppressed the protein production of FGF2 in sinonasal mucosa of CRS.
**G-CSF**	Matsuoka *et al* [22]	CAM suppressed the production of G-CSF by IL-1β-added human synoviocytes obtained from RA and OA patients.
**GM-CSF**	Matsuoka *et al* [22]	CAM suppressed the production of GM-CSF by IL-1β-added human synoviocytes obtained from RA and OA patients.
	Zeng *et al* [29]	Anti-inflammatory effect of CAM on ethmoid mucosal tissue of CRS was investigated. CAM suppressed the protein production of GM-CSF in sinonasal mucosa of CRS patients.
**MMP-9**	Sassa* et al* [61]	Rat mammary adenocarcinoma cells were treated with CAM, and total RNAs were extracted from tumour cells at 6, 12, 24, 48, and 72 hours. CAM inhibited the expression of MMP-9 gene in rat mammary adenocarcinoma cells.
	Zeng *et al* [29]	Anti-inflammatory effect of CAM on ethmoid mucosal tissue of CRS was investigated. CAM suppressed the protein production of MMP-9 in sinonasal mucosa of CRS patients.
	Zimmermann *et al* [30]	The immunomodulatory effects of macrolides (review of literature).
	Takahashi *et al* [71]	CAM suppressed induction of MMP-9 in mouse monocytes and improved pathological changes in the lung and heart of mice infected with influenza A virus.
**Cytokines enhanced by CAM**		
**Cytokines**	**References**	**Supplementary explanations**
**IL-4**	Hamada *et al* [36]	LLC-bearing mice received anticancer chemotherapy after tumour inoculation, and CAM treatments (for 7 days) started immediately after the chemotherapy or 1 week later after the chemotherapy (i.e., delayed initiation of CAM treatment). The mice were sacrificed after finishing CAM treatments. The tumour growth and the number of IL-4-producing T-cells in the spleen were evaluated. The delayed initiation of CAM treatment was effective for reducing tumour growth and resulted in increased IL-4-producing T-cells.
IL-10*	Morikawa *et al* [23]	CAM enhanced the synthesis of IL-10 by LPS-stimulated healthy human monocytes.
	Zeng *et al* [29]	Experiment using ethmoid mucosal tissue from CRS showed that CAM enhanced the protein production of IL-10 in sinonasal mucosa of CRS.
IL-12*	Teramoto *et al* [53]	Patients with inoperable NSCLC were treated with CAM. The level of IL-12 mRNA was elevated after CAM treatment.
	Majima *et al* [54]	Patients with unresectable NSCLC were treated with CAM for 3 months. The expression of IL-12 mRNA was measured during CAM treatment. The expression of IL-12 mRNA significantly increased during the treatment.
	Majima *et al* [44]	Patients with advanced NSCLC were treated with CAM. The expression of IL-12 mRNA in PBMNCs obtained from the patients was measured during CAM treatment. In an additional experimental model, mice were inoculated subcutaneously with LLC cells and treated with CAM for 1 week. Thereafter, the mice were sacrificed, and the expression of IL-12 mRNA in spleen cells was evaluated. The expression of IL-12 mRNA in PBMNCs and mouse spleen cells increased after CAM treatments.
	Majima *et al* [55]	Patients with NSCLC were treated with CAM for 3 months. The expression of IL-12 mRNA in PBMNCs increased during CAM treatment.
IFN-γ*	Majima *et al* [54]	Patients with unresectable NSCLC were treated with CAM. The expression of IFN-γ mRNA in PBMNCs was evaluated during CAM treatment. The expression of IFN-γ mRNA significantly increased during the treatment.
	Majima *et al* [44]	Patients with advanced NSCLC were treated with CAM. The expression of IFN-γ mRNA in PBMNCs was evaluated during the treatment. In an additional experimental model, mice were inoculated subcutaneously with LLC cells and treated with CAM for 1 week. Thereafter, the mice were sacrificed, and the expression of IFN-γ mRNA in spleen cells was evaluated. The expression of IFN-γ mRNA in PBMNCs and mouse spleen cells increased after CAM treatments.
	Majima *et al* [55]	The patients with advanced NSCLC were treated with CAM for 3 months. The expression of IFN-γ mRNA in PBMNCs increased during CAM treatment.
	Hamada *et al* [36]	LLC-bearing mice received anticancer chemotherapy after tumour inoculation, and CAM treatments (for 7 days) started immediately after the chemotherapy or 1 week later after the chemotherapy (i.e., delayed initiation of CAM treatment). The mice were sacrificed after finishing CAM treatments. The tumour growth and the number of IFN-γ-producing T-cells in the spleen were evaluated. The delayed initiation of CAM treatment was effective in reducing IFN-γ-producing T-cells.

* The influence of CAM on IL-10, IL-12 and IFN-γ showed conflicting results.

Abbreviations: BOOP, bronchiolitis obliterans organising pneumonia; CRS, chronic rhinosinusitis; mRNA, messenger RNA; NSCLC, non-small cell lung cancer; RA, rheumatoid arthritis; OA, osteoarthritis; LLC, Lewis lung carcinoma; MM, multiple myeloma; LPS, lipopolysaccharide; PBMNC, peripheral blood mononuclear cell; EBC, exhaled breath condensate.
